# Study on the Absorption and Immunity Regulation of Simulated Breast Milk Nutrients in Rats

**DOI:** 10.3389/fnut.2022.769846

**Published:** 2022-03-15

**Authors:** Qinggang Xie, Jinlan Zhang, Yang Liu, Yi Yang, Yanli Wei, Shilong Jiang, Dongying Cui, Qile Zhou

**Affiliations:** ^1^Heilongjiang Feihe Dairy Co., Ltd., Beijing, China; ^2^Beijing Institute of Nutritional Resources, Beijing Academy of Science and Technology, Beijing, China

**Keywords:** simulated-human milk nutrients, immunity, absorption *in vivo*, vitamin, mineral

## Abstract

Since infant formula (IF) manufacturers aim to produce a product as close to breast milk as possible, fortified nutrients are usually added. Generally, an IF is produced by adjusting the types and proportions of vitamins and minerals. This study comparatively examined the content of the six nutrients in different compound forms *in vivo* and evaluated the effect of different nutrient pack groups on immunity and growth. The results indicated that the simulated-human milk nutrients [minerals zinc (Zn), iron (Fe), calcium (Ca), and vitamins A, E, and B_1_] were more easily absorbed by the body while effectively regulating immunity. This study provides a scientific foundation for developing, manufacturing, and applying imitation-breast formula milk powder.

## Introduction

Healthy infants represent the future of any country. The age of 0–12 months denotes the rapid development period of infants and children and signifies the critical period of life. Breast milk is considered the ideal food for neonates and is the preferred diet for preterm infants and those with an exceedingly low birth weight (1–3). Since breast milk contains abundant immunological constituents, such as immunoglobulins and lysozymes, that can protect against viruses (4), it is crucial for infant growth. However, some infants cannot be exclusively breastfed. Since pure liquid milk is deficient in certain nutrients and does not meet the nutritional requirements of babies, it is unsuitable for direct consumption by infants. Therefore, liquid milk is mixed with additional nutrients to form a powdered formula more appropriate for the needs of infants according to the corresponding age. To render infant formula (IF) more similar to the breast milk, IF manufacturers have added ingredients, such as long-chain polyunsaturated fatty acids, nucleotides, prebiotics, probiotics, and adjusted the form types and proportions of vitamins and minerals (5). Novel ingredients and new techniques in the dairy industry can minimize the difference between breast milk and formula milk (6).

Although IF is relatively close to breast milk, it cannot completely replace the nutritious value of breast milk, necessitating the inevitable addition of nutrients during IF production. However, the raw materials used in IF are strictly regulated. The original, commonly used nutrient fortified package is adjusted in this study to improve product quality and render it closer to breast milk. The manufacturing and nutritional content of IFs are highly regulated to ensure that growing and developing infants who are not breastfed receive adequate nutrition (7). The vitamins and minerals in formula milk powder are crucial for infant growth and immunity and typically consist of one or more combinations. For example, zinc (Zn)-containing compounds that can be used in IF are Zn sulfate, Zn gluconate, Zn oxide, Zn lactate, Zn citrate, Zn chloride, and Zn acetate, the ratios, and content of which are essential for optimal infant growth (8), because elements in different conjugate forms have different digestibility and absorption *in vivo*. Besides, the strengthening vitamins and minerals of IF in China are needed to meet the requirements of GB 14880 (National food safety standard Food nutrition enhancer use standard).

The bioavailability of vitamins and minerals in IF is mainly affected by synergistic and antagonistic interactions of the components. The bioavailability of non-heme iron is relatively lower than that of heme iron (≈10% instead of ≈25%) (9). Meanwhile, vitamins A, C, and E and folic acid promote iron absorption (10). Therefore, selecting the appropriate compound to improve the bioavailability of vitamins and minerals is vital. This study investigated the *in vivo* absorption of three vitamins (vitamins A, E, and B_1_) and three minerals [Zn, iron (Fe), and calcium (Ca)] of different compound forms and their relationship with immunity, growth, and development. Although the previous nutrient-enhanced formula package, *Feihe Dairy*, used vitamin A acetate (V-A), dl-alpha-tocopheryl acetate (V-E), thiamine mononitrate (V-B_1_), Fe pyrophosphate, Zn sulfate, and Ca carbonate, their bioavailability was not satisfactory. Furthermore, the side effects of ferrous sulfate were significantly higher than ferric pyrophosphate, while Zn sulfate irritated the gastrointestinal tract of infants (11, 12). Consequently, a new nutrient formula was developed and investigated, which was closer to the ingredient composition of breast milk, and included vitamin A palmitate, d-α-tocopheryl acetate, thiamine hydrochloride, Zn gluconate/Zn citrate, Fe gluconate, and Ca citrate.

In this study, weaning Sprague-Dawley (SD) rats were fed the previous and new nutrient packages (containing different compound forms of V-A, E, and B_1_, and Zn, Fe, and Ca) to comparatively analyze the content of six nutrients *in vivo* and to evaluate the effect of the different nutrient packages on the immunity and growth of the respective groups. This study provides a scientific foundation for developing imitation-breast formula milk powder and its application during IF manufacturing.

## Methods, Materials, and Instruments

### Animal Experiment

A total of 56 weaning male SD rats, each with an initial body weight (BW) of 65.3 ± 4.35 g, were provided by the Beijing Vital River Laboratory Animal Technology Co. Ltd. (Beijing, China). The rat feed for each experimental group was customized and provided according to the nutrient formula prescribed by the Beijing HFK Bio-Technology Co. Ltd. The rats were housed individually in standard cages that allowed food intake recording. The cages were placed in a temperature- and humidity-controlled room (25 ± 2°C, 55 ± 2% humidity) with a 12 h/12 h light/dark cycle (the light was switched off at 8:00 pm). The rats were allowed free access to water and food, acclimatized for 3 days, and fed the control diet prior to the experiment. Animal welfare and experimental procedures were approved by the Beijing Normal University Laboratory Animals Care and Use Committee (no. SCXK2019-0008) in accordance with the guide for the care and use of laboratory animals.

The rats were randomly assigned to seven groups (nos. 1–7) of equal size. The control group was fed a basic diet without a nutrient package, while the remaining six groups received different forms and quantities of vitamins and minerals (Table 1). The animal experiment was performed for five consecutive weeks. In this period, blood was collected from the orbital venous plexus of each rat in different groups at days 0, 7, 14, 21, 28, and 35, then the serum immune factors, i.e., interleukin-2 (IL-2), interleukin-4 (IL-4), interferon-γ (IFN-γ), immunoglobulin M (IgM), immunoglobulin A (IgA), and immunoglobulin G (IgG), were analyzed. Meanwhile, the weight and feed consumption of the rats were recorded weekly. After a feeding period of 5 weeks, the rats were anesthetized and then killed by cervical dislocation, and blood was taken from the heart to determine the vitamin A, E, and B_1_ levels in the rat serum. Then the livers, kidneys, and femurs were dissected immediately, weighed, and recorded. All the biological samples were stored in a freezer at −80°C until subsequent Ca, Fe, and Zn mineral analysis.

**Table 1:** Grouping and dosing of experimental animals.

**Composition**	**Dietary treatments**						
	**Group1: Basic feed group**	**Group2: Previous nutrient fortified package**	**Group3: New nutrient fortified package-L**	**Group4: New nutrient fortified package-M**	**Group5: New nutrient fortified package-H**	**Group6: New nutrient fortified package-C**	**Group7: New nutrient fortified package-MC**
Crude Protein (g/100 g)	19.20	19.20	19.20	19.20	19.20	19.20	19.20
Crude Fat (g/100 g)	4.60	4.60	4.60	4.60	4.60	4.60	4.60
Crude Fiber (g/100 g)	4.00	4.00	4.00	4.00	4.00	4.00	4.00
Carbohydrate (g/100 g)	55.90	55.90	55.90	55.90	55.90	55.90	55.90
Crude Ash (g/100 g)	6.30	6.30	6.30	6.30	6.30	6.30	6.30
Moisture (g/100 g)	8.80	8.80	8.80	8.80	8.80	8.80	8.80
Methionine (g/100 g)	0.45	0.45	0.45	0.45	0.45	0.45	0.45
Lysine (g/100 g)	1.11	1.11	1.11	1.11	1.11	1.11	1.11
Cystine (g/100 g)	0.64	0.64	0.64	0.64	0.64	0.64	0.64
Vitamins (mg/100 g)							
Vitamin A acetate	-	4.50	-	-	-	-	-
Vitamin A palmitate	-	-	6.00	12.00	18.00	12.00	12.00
dl-α-tocopheryl acetate	-	26.10	-	-	-	-	-
d-α-tocopheryl acetate	-	-	27.80	55.60	83.30	55.60	55.60
Thiamine mononitrate	-	1.04	-	-	-	-	-
Thiamine hydrochloride	-	-	1.10	2.30	3.40	2.30	2.30
Minerals (mg/100 g)							
Zinc sulfate	-	12.50	-	-	-	-	-
Zinc gluconate	-	-	31.50	63.00	94.40	-	31.50
Zinc citrate	-	-	-	-	-	84.5	42.30
Iron pyrophosphate	-	20.00	-	-	-	-	-
Ferrous gluconate	-	-	43.50	87.00	130.40	87.00	87.00
Calcium carbonate	-	1,000.00	-	-	-	-	-
Calcium citrate	-	-	1,900.00	3,810.00	5,710.00	3,810.00	3,810.00

### Determination of Vitamins A, E, and B_1_

The three vitamins were determined by ultra-high-performance liquid chromatography (UHPLC, Thermo Fisher Scientific Corporation) and it was conducted using Accucore Vanquish, consisting of a VH-P10 high-pressure dual system pump, a VH-A10 automatic sampler, an H column oven, and an HL diode array detector. Data acquisition was performed using the Chromeleon 7.2 SR Data Processing System. Other instruments included a BS-110S electronic balance (Beijing Sartorius Scales Co., Ltd., China) and a 5424R high-speed tabletop centrifuge (Eppendorf Ltd., Germany). The acetonitrile and methanol were of chromatographic grade and were supplied by Fisher Scientific, USA. High-purity water was obtained using a Milli-Q Advantage ultra-pure water system.

For chromatographic separation, the contents of vitamins A and E were determined using an Agilent ZORBAOX SB-C18 column (250 × 4.6 mm, 5 μm, Agilent Technology Co. Ltd., USA). The mobile phase consisted of water (A) and methanol (B). The gradient elution conditions were optimized as follows: 96% B (0–3 min), 96–100% B (3–20 min), 100% B (20–24 min), 100–96% B (24–24.5 min), and 96% B (24.5–30 min) at a flow rate of 0.8 ml/min. The temperatures of the column and autosampler were maintained at 25 and 4°C, respectively. The detection wavelengths were 325 nm for vitamins A and 294 nm for vitamins E, while a 10 μl injection volume was used for the reference standards and samples.

The vitamin B_1_ content in the rat serum was determined using a Thermo Scientific Hypersil GOLD aQ column (250 × 4.6 mm, 5 μm, Thermo Fisher Scientific Ltd., USA). The mobile phase consisted of 0.05 mol/L sodium acetate in A and B (V/V: 65/35) at a 0.8 ml/min flow rate. The temperatures of the column and autosampler were maintained at 25 and 4°C, respectively. The excitation wavelength was set at 375 nm, while the emission wavelength was 435 nm. The serum sample pretreatment for vitamins A and E was performed according to the available literature (13, 14).

### Determination of Minerals Zn, Fe, and Ca

The Ca, Fe, and Zn levels were measured using a PinAAcle 900T flame atomic absorption spectrometer (Perkin Elmer Ltd., USA) and the MARS Classic 6 automatic high throughput microwave digestion and extraction system (PYNN Ltd., USA). Nitric acid (65%) was obtained from Merck, Germany. The mineral standards were obtained from the National Certified Reference Materials Resource Center.

The sample pretreatment for elemental analysis was occurred as follows: 8 ml HNO_3_ was added to an accurately weighed tissue sample of 0.4 g and subjected to a preprogrammed microwave digestion procedure. Next, the mixture was cooled, and the excess HNO_3_ was evaporated on a hot plate set at 160°C and transferred quantitatively to a volumetric flask after cooling the digestion tube. The sample was then diluted according to actual measurement requirements and mixed well for subsequent analysis. The parameters and reference conditions for the mineral analysis included wavelengths of 422.7 (Ca), 248.3 (Fe), and 213.9 (Zn), slit widths of 1.3 (Ca) and 0.2 (Fe and Zn), light currents of 5–15 mA (Ca and Fe) and 3–5 mA (Zn), a burner height of 3 mm, an airflow rate of 9 L/min, and an acetylene flow rate of 2 L/min for Ca, Fe, and Zn (15).

### ELISA Analyses of the Immunity-Related Indicators

ELISA was performed using an enzyme-labeled instrument (Labsystems Multiskan MS, Finland) and an AC8 Microplate Washer (Thermo Labsystems, Finland). Other instruments included a GNP-9080 water-jacket thermostatic incubator (Shanghai Kuncheng Scientific Instrument Co., Ltd), Mettler XS105DU electronic balance (Mettler Ltd., Switzerland), T16W high-speed micro-centrifuges (Changzhou Jintan Liangyou Instrument Co., Ltd., China), and Potter-Elvehjem tissue grinders. The bicinchoninic acid (BCA) protein assay kit and ELISA kits for IL-2, IL-4, IFN-γ, IgA, IgG, and IgM were purchased from Meimian Biotechnology Co. Ltd. (Nanjing, Jiangsu Province, China).

The ELISA procedure was performed as follows: each concentration of the standard sample was measured, 50 μl/well, for parallel control. Next, 50 μl of the specimen consisting of 40 μl of the diluted sample liquid and 10 μl of the sample was loaded into each well, while 50 μl of the diluted standard liquid or sample liquid was added into the blanks. The standard and test sample additions were completed in 15 min, covered with a plate sealing membrane, and incubated at 37°C for 30 min. Next, 300 μl detergent was added, after which the plate was placed on a vortex generator for 5 s. Enzyme reagent (50 μl) was added and incubated for 30 min to induce an additional color reaction, after which the results were obtained using a plate reader. Preparation of standard curve was done to achieve a serial dilution of the standard sample with the concentration from high to low. The IL-2, IL-4, IFN-γ, IgA, IgG, and IgM concentrations in the plasma were then analyzed using an ultrasensitive rat ELISA kit according to the instructions.

### Statistical Analysis

The data were analyzed via a Student's *t*-test and one-way ANOVA using the StatView Statistics software program (Brainpower, Calabasas, CA, USA) to determine the constant variance and normal distribution patterns. The results that did not fit these conditions were transformed to valid data using logarithms or square roots. The data were presented as means ± SEM. Differences were considered significant when *p* < 0.05 and were represented as ^*^ for *p* < 0.05, ^**^ for *p* < 0.01, and ^***^ for *p* < 0.001.

## Results and Discussion

### Investigating the Different Forms of the Six Nutrients *in vivo*

Minerals and trace elements account for about 4% of the total human body mass and are crucial in preserving the bone structure, regulating certain bodily functions, and maintaining the water balance in the body (16, 17). Vitamins also play a vital role in normal life activities. Therefore, it is essential that IF products intended for infants contain minerals and vitamins in amounts that satisfy their nutritional requirements (18).

**Figure 1:**
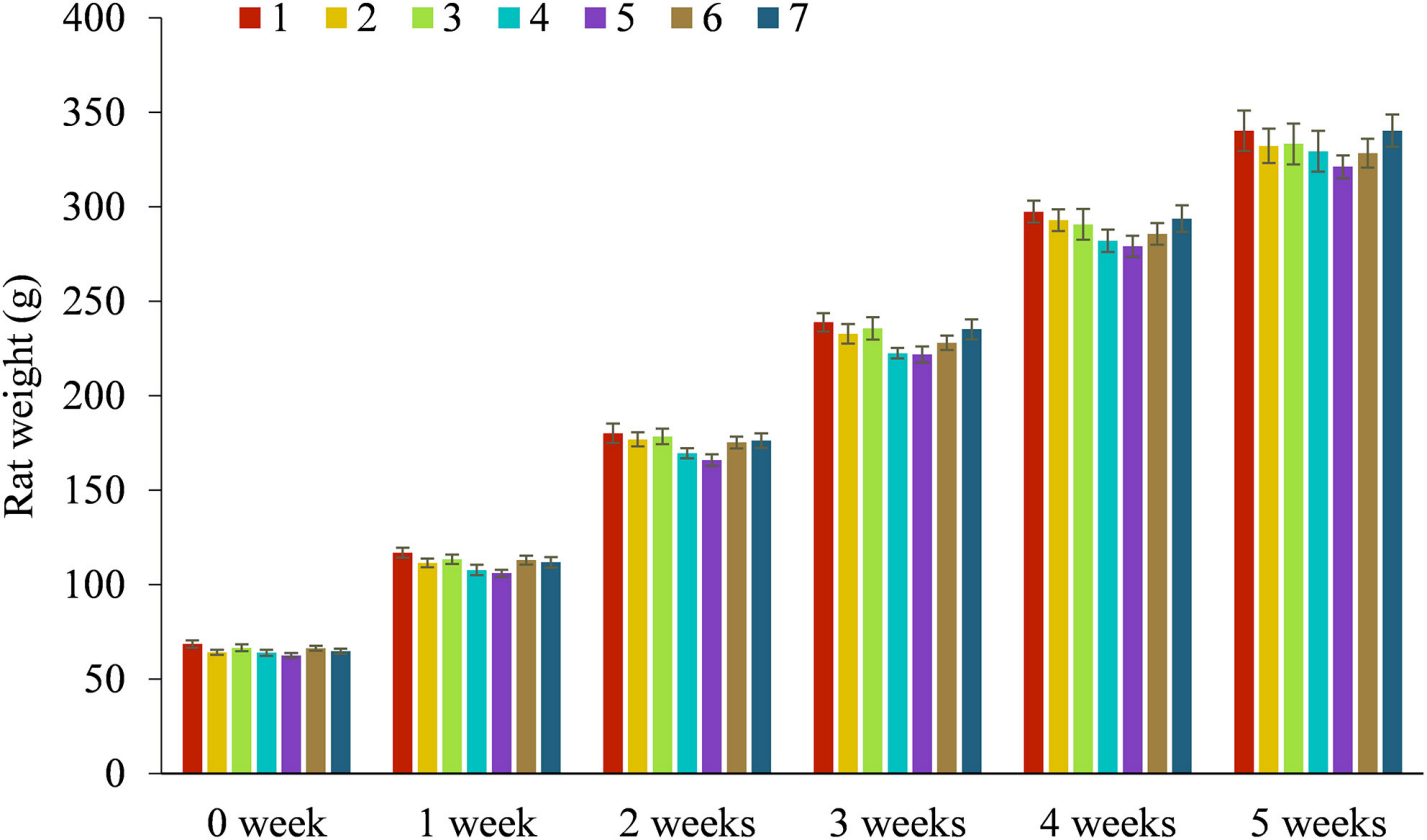
Effect of body weight trends in SD rats treated with groups 1–7 in five consecutive weeks, *n* = 8 per each group. 1: Blank control, 2: former type, 3: new-L, 4: new-M, 5: new-H, 6: new-C, 7: new-MC.

During the 5-week experimental period, the BW of the rats increased continuously in the seven groups, while no differences were evident between the groups (Figure 1). The vitamins A, E, and B_1_ content in the rat serum and the Zn, Fe, and Ca levels in the livers, kidneys, and femurs were analyzed, which are listed in Table 2. The results showed that the content levels of the six test nutrients were distinctly higher in all the new package groups (3–7) than in the former type (group 2). This indicated that the simulated human milk nutrients (such as vitamin A palmitate and Ca citrate) were more easily absorbed *in vivo* than the standard types (such as vitamin A acetate and Ca carbonate), even though the total content levels of the six nutrients in the rat feed of groups 2 and 3 were exactly the same. Furthermore, the results obtained from groups 3–5 indicated that the levels of all the *in vivo* nutrient levels increased in conjunction with a higher administered dose, confirming dose dependency. Moreover, comparing the nutrients in the fortified packages with the dietary intake references showed that the nutrient intake levels of all the groups met the dietary reference intakes (DRIs) requirements (19) and were below the tolerable upper intake level (UL). Regarding groups 4 (Zn gluconate), 6 (Zn citrate), and 7 (Zn gluconate: Zn citrate = 1:1), the content of Zn in group 6 was higher than that of 7 and 4, indicating a rationale for the presence of Zn citrate in human milk. The greater bioavailability of Zn species used for fortification can be crucial. Studies have shown that zinc citrate is a form of zinc in breast milk and zinc bioavailability in human milk is for infant much higher than in cow's milk (20). In addition, the advantage of zinc citrate over zinc sulfate is related to its better sensory qualities (21). Meanwhile, the contents of the 6 nutrients in group 3 (closer to the ingredients of breast milk) were higher than that of group 2 in the same dose, which confirmed the reason why breast milk is the natural ideal food for infants.

**Table 2:** The contents of tested vitamins A, E, B_1_, and minerals Ca, Fe, Zn *in vivo*.

**Group**	**Control 1**	**Former type 2**	**New-L 3**	**New-M 4**	**New-H 5**	**New-C 6**	**New-MC 7**
Contents of **Vitamin A** in rat serum (μg/ml)							
Mean ± SE	1.42 ± 0.148^**^	2.34 ± 0.109	2.84 ± 0.151	3.25 ± 0.175^**^	3.51 ± 0.107^***^	3.33 ± 0.166^***^	3.39 ± 0.211^***^
Contents of **Vitamin E** in rat serum (μg/ml)							
Mean ± SE	0.40 ± 0.040^***^	1.37 ± 0.088	1.52 ± 0.071	2.10 ± 0.157^**^	2.52 ± 0.104^***^	2.17 ± 0.229^***^	2.15 ± 0.207^***^
Contents of **Vitamin B**_**1**_ in rat serum (μg/ml)							
Mean ± SE	34.90 ± 4.19^*^	76.46 ± 6.85	92.13 ± 9.43	148.69 ± 13.22^***^	195.38 ± 13.81^***^	153.32 ± 14.13^***^	151.66 ± 12.76^***^
Contents of **Ca** in rat femurs (mg/g)							
Mean ± SE	3.95 ± 0.256^**^	4.87 ± 0.202	5.38 ± 0.244	5.77 ± 0.237^**^	6.25 ± 0.311^***^	5.96 ± 0.278^***^	5.80 ± 0.312^**^
Contents of **Fe** in rat liver^&^ and kidney^#^ (μg/g)							
Mean ± SE^&^	30.52 ± 1.33^*^	43.27 ± 2.41	50.56 ± 4.03	59.29 ± 5.63^*^	64.15 ± 5.45^**^	60.45 ± 5.74^**^	61.21 ± 4.46^**^
Mean ± SE^#^	27.39 ± 1.05	33.04 ± 1.57	38.12 ± 2.66	42.18 ± 2.43^**^	47.33 ± 3.23^***^	43.29 ± 2.50^**^	43.19 ± 1.78^**^
Contents of **Zn** in rat liver^&^ and kidney^#^ (μg/g)							
Mean ± SE^&^	17.26 ± 1.05^*^	22.68 ± 1.74	26.87 ± 1.37	29.51 ± 1.34^**^	35.12 ± 1.84^***^	32.82 ± 2.38^***^	31.35 ± 1.99^**^
Mean ± SE^#^	18.94 ± 1.29	22.67 ± 1.53	24.39 ± 1.56	26.92 ± 1.26	28.34 ± 1.64^*^	29.53 ± 2.03^**^	28.45 ± 1.58^*^

* *indicates p < 0.05*,

** *indicates p < 0.01*,

*** *indicates p < 0.001 vs. group 2 (Former type)*.

# *indicates the contents of Fe or Zn in rat liver*

& *indicates the contents of Fe or Zn in rat kidney*.

### The Connection Between Nutrients and Immunity

Regarding the immunology research, immunoglobulin and cytokine secretion levels in five consecutive weeks were evaluated. IL-2 and INF-γ mediate cellular immunity. IL-2 stimulates the proliferation of activated B cells to secrete immunoglobulin while inducing the growth and differentiation of T cells to produce IFN-γ. Moreover, IL-2 enhances the cytotoxicity of T lymphocytes and natural killer cells. INF-γ activates macrophages to swallow antigens and kills pathogenic microorganisms specifically. IL-4, mainly produced by activated T nuclear monocytes, stimulates the proliferation of B cells, promotes and regulates Ig E production, stimulates T cell growth, and encourages many inflammatory reactions (22).

**Figure 2:**
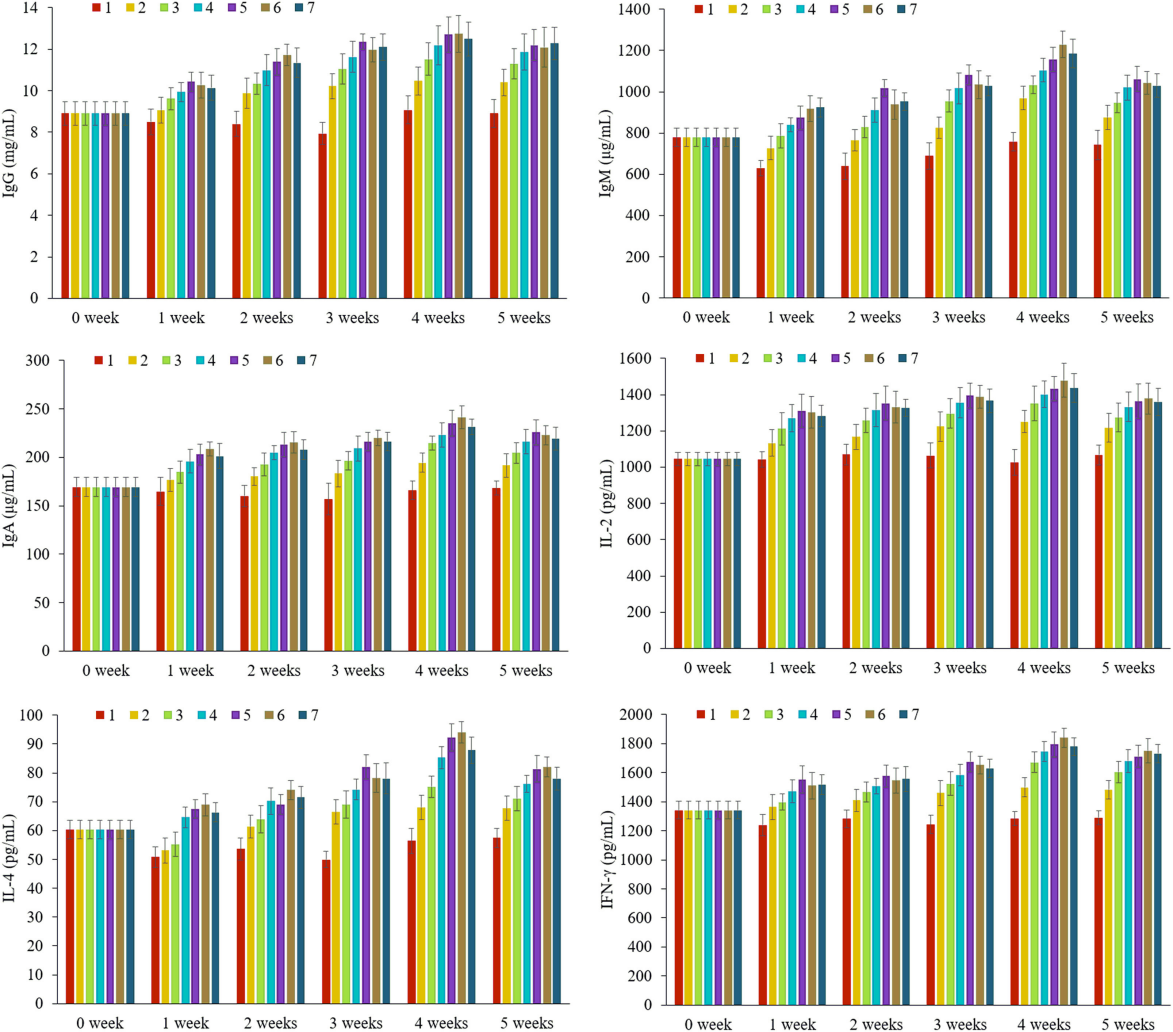
The contents of tested immunoglobulin M (IgM), immunoglobulin G (IgG), immunoglobulin A (IgA), interleukin (IL)-2, IL-4, and interferon-γ (IFN-γ) in different groups at 0–5 weeks, *n* = 8 per each group. 1: Blank control, 2: former type, 3: new-L, 4: new-M, 5: new-H, 6: new-C, 7: new-MC.

As shown in Figure 2, the IL-2, IL-4, IFN-γ, IgA, IgG, and IgM levels in the rat serum are examined in 0–5 weeks (Supplementary Table S1). It was obvious that the levels of each immunoglobulin and cytokine showed an increasing trend from 0 to 5 weeks except for group 1 and reached the peak value in the fourth week, then decreased slightly in the fifth week. Take the secretion level of immunoglobulin as an example, immunoglobulin is a kind of protein with antibody activity, and the level of immunity can be reflected by the content of immunoglobulin in serum. The results illustrated that with the extension of feeding time, the immune function of each group supplemented with nutrients was continuously enhanced, which could significantly increase the levels of immunoglobulins in the blood. The slight decrease in the fifth week might be that the nutrient package had reached the absorption threshold, suggesting that excessive intake of nutrients would weaken the immune response.

In order to obtain a suitable immune response, it is necessary to supply sufficient and balanced nutrients. Different physiological stages need different elements, which is caused by the differences in bioavailability, metabolic level, and loss degree. For group 1 (contained no nutrient package), the contents of immunoglobulins and cytokines during the growth and development of 1–5 weeks were mostly lower than the level of weaning rats at 0 week, which indicated that the lack of trace elements and vitamins could lead to nutritional dysfunction and would reduce the immune cells function and body immunity. However, for group 2 (basic nutrient package group), the contents of immunity indicators began to increase from the first or second week of feeding until the fifth week, while in other groups 3–7, the levels of immunity factors increased significantly at the first week of feeding, which were higher than the levels of newborns at 0 week, indicating that immunity was significantly improved. Comparing the old and new nutrient packages (groups 2 and 3), we found that the immunoglobulin contents of group 3, which were closed to the constituents of breast milk, were significantly higher than that of group 2, while the contents of vitamins and minerals in group 3 were also higher than those of group 2, indicating that the simulated-human milk nutrients were more easily absorbed, and the improvement of immunity has a certain proportional relationship with the contents of these six elements in the body. For groups 3–5, we found that the contents of immunity indicators were dose dependent with low, medium, and high concentration. Comparing groups 4, 6, and 7 in different forms but the same dose of Zn, we found that the contents of immunity indexes in rats were 6 > 7 > 4, and the contents of immunoglobulins in groups 6 and 7 even exceeded that of the high-dose group (5), which indicated that zinc citrate was more beneficial to the absorption in the body, this was consistent with the clinical experiment conducted by Xiao Mei and others in 2008 (23).

Furthermore, we focused on the immunity-related data in the fourth week as each immunity indicator reached the peak and optimal. As shown in Table 3, the contents of the 6 indexes in group 1 are significantly lower than in the other groups (*p* < 0.01, vs. groups 2–7), indicating the importance of fortified nutrients for immunity. From the results of groups 3–5, although displayed dose dependence, but the high-dose group was not obvious in some indexes, such as IgG, this might be attributed to that the nutrients of the middle-dose group could meet the requirements for growth and immunity. A comparative analysis between groups 4, 6, and 7 revealed that the content levels of the six indexes in groups 6 and 7 even surpassed the high-dose group, confirming that Zn citrate was more conducive to absorption and immune enhancement. Nutrient deficiency reportedly affects innate and adaptive immune responses and the number of B cells (24, 25). The results indicated that the nutrient-fortified package could promote the level of immune factors in the body, enhancing immunity.

**Table 3:** The contents of tested immunoglobulin M (IgM), immunoglobulin G (IgG), immunoglobulin A (IgA), interleukin (IL)-2, IL-4, and I interferon-γ (FN-γ) in groups 1–7 at fourth week (Mean ± SE, *n* = 8).

**Group**	**IgG (mg/ml)**	**IgA (μg/ml)**	**IgM (μg/ml)**	**IL-2 (pg/ml)**	**IL-4 (pg/ml)**	**IFN-γ (pg/ml)**
1	9.07 ± 0.66	165.93 ± 9.26	758.11 ± 43.73	1027.15 ± 72.09	56.39 ± 4.29	1283.95 ± 47.09
2	10.47 ± 0.68	194.53 ± 10.06	970.02 ± 58.52	1251.94 ± 61.73	68.04 ± 4.12	1499.24 ± 66.79
3	11.52 ± 0.77	214.84 ± 7.23	1032.67 ± 42.84	1352.29 ± 96.79	75.23 ± 3.73	1672.13 ± 73.08
4	12.20 ± 0.94	223.32 ± 12.46	1104.94 ± 56.50	1402.26 ± 73.71	85.29 ± 3.78	1746.27 ± 70.27
5	12.70 ± 0.86	235.14 ± 13.06	1157.74 ± 58.49	1433.91 ± 67.60	92.24 ± 4.68	1794.31 ± 86.11
6	12.74 ± 0.89	241.49 ± 11.94	1229.82 ± 64.47	1479.56 ± 93.06	94.01 ± 3.70	1841.78 ± 65.49
7	12.49 ± 0.82	231.55 ± 8.03	1184.57 ± 69.68	1436.90 ± 77.65	87.82 ± 4.59	1780.76 ± 60.93

Lack of one or more nutrients can weaken immune function and increase susceptibility to infectious pathogens. This is probably because these nutrients are involved in the response of molecules and cells to immune system attacks. The supplement of specific nutrients can restore immune function and increase the body anti-infectivity, which reflect the intrinsic relationship between nutrient intake and immune function.

Breast milk contains immune components, such as immunoglobulins, immune cells, lactoferrin, and lysozymes (26). However, the content of active immune factors in IF is limited. Some IF nutrients are related to the human immune system, such as vitamin A, Fe, and Zn. Vitamin A is essential for visual development and cell differentiation while assisting with immune system establishment (27, 28). Lactoferrin can also transport Fe as an immune factor, while Zn is vital for immune cell functionality and cytokine secretion. Furthermore, studies have shown that cooperation between nutrients, such as vitamin A and Zn, has a synergistic effect on immunity. IF products are generally manufactured by combining different ratios of fortified nutrients (29). Some IF products contain different ratios of insoluble and soluble Ca salts to achieve the desirable Ca levels (e.g., chlorine and phosphate) (26, 30). The result of this study revealed a relatively higher immune level at a 1:1 ratio of Zn citrate to Zn gluconate.

The nutritional types and additions to the new and old nutrient fortified packages satisfied the policy requirements. However, different kinds of nutrients present differences in taste, absorption, and process stability. Replacing or adding nutrients to IF requires various considerations. The ultimate goal is for infants to achieve adequate growth and development while enhancing their immunity (29, 31, 32).

## Conclusions

This paper investigates six nutrients (V-A, E, B_1_, Zn, Fe, and Ca) in different compound forms. Weaning SD rats are fed the original and new nutrient packages, after which the content levels of the six nutrients are analyzed *in vivo* while evaluating their effect on immunity and growth. According to the weight and growth of rats in 0–5 weeks, as shown in Figure 1, the doses of each group have no significant adverse effect on rats. The results show that the content levels of the target nutrients in the serum, livers, kidneys, bone of the rats in group 3 are significantly higher than in group 2, indicating that the simulated breast milk nutrients (vitamin A palmitate, d-α-tocopheryl acetate, thiamine hydrochloride, Zn gluconate, Zn citrate, Fe gluconate, and Ca citrate) are more easily absorbed *in vivo*. Moreover, the immunity expression of groups 3–7 is superior to group 2, demonstrating that the simulated breast milk nutrients can help to regulate or enhance immunity. Furthermore, Zn citrate is more successful in regulating immunity than Zn gluconate, as shown by comparing groups 4, 6, and 7.

Since it is very hard to examine the bioavailability and the immune responses of nutrients in human infants, we utilize animal models to clarify the effects of six nutrients in different compound forms *in vivo*. In the rat study, it is obvious that an adequate and balanced supply of these nutrients is necessary if a suitable immune response is to be obtained. Lack of one or more nutrients can weaken immune function. This study provides new insights into how compound-form and quantity of nutrients can differ in their effects on the bioavailability and immune function.

In summary, a comparison of vitamins and minerals absorptions and immunity effect in rats suggests that the formula provided to groups 6 and 7 can be used as an appropriate nutritional fortification. The information gained from the rat results enables the re-design of IF formulations to deliver human milk-like benefits. It also lays a scientific foundation for the research and development and quality improvement of the vitamins and minerals fortification. Further research is needed to elucidate the mechanism by which the combination of the specific six nutrients improves the bioavailability and immune system.

## Data Availability Statement

The original contributions presented in the study are included in the article/Supplementary Material, further inquiries can be directed to the corresponding authors.

## Ethics Statement

The animal study was reviewed and approved by the AAALAC (Association for Assessment and Accreditation of Laboratory Animal Care International), and the IACUC (Institutional Animal Care and Use Committee) of Peking University.

## Author Contributions

QX, JZ, DC, and QZ designed the study and manuscript writing. QX, JZ, and YL did the laboratory work in the expression and statistical analysis. YY participated in the data analysis and the revision of articles critically for important intellectual content. YW, SJ, and DC contributed to data analysis and interpretation. All the authors read and approved the manuscript.

## Funding

This research was funded by Technology Major Special Project of Heilongjiang Province: Dairy Products and Meat Processing (No. 2020ZX07B01), Beijing Municipal Excellent Talents Foundation (2018400685627G341), Fengtai Nova Program (KJXX201904), and Beijing Postdoctoral Research Foundation.

## Conflict of Interest

QX, YL, SJ, and DC were employed by the company Heilongjiang Feihe Dairy Co., Ltd. The remaining authors declare that the research was conducted in the absence of any commercial or financial relationships that could be construed as a potential conflict of interest.

## Publisher's Note

All claims expressed in this article are solely those of the authors and do not necessarily represent those of their affiliated organizations, or those of the publisher, the editors and the reviewers. Any product that may be evaluated in this article, or claim that may be made by its manufacturer, is not guaranteed or endorsed by the publisher.

## References

[1] Agostoni C Braegger C Decsi T Kolacek S Koletzko B Michaelsen KF. Breast-feeding: a commentary by the ESPGHAN committee on nutrition. J Pediatr Gastr Nutr. (2009) 49:112–25. 10.1097/MPG.0b013e31819f1e05 19502997

[2] Agostoni C Buonocore G Carnielli VP De Curtis M Darmaun D Decsi T. Enteral nutrient supply for preterm infants: Commentary from the European society of paediatric gastroenterology, hepatology and nutrition committee on nutrition. J Pediatr Gastr Nutr. (2010) 50:85–91. 10.1097/MPG.0b013e3181adaee0 19881390

[3] Eidelman AI. Breastfeeding and the use of human milk: an analysis of the American Academy of Pediatrics 2012 Breastfeeding Policy Statement. Breastfeed Med. (2012) 7:323–4. 10.1089/bfm.2012.0067 22946888

[4] Mosca F Giannì ML. Human milk: composition and health benefits La Pediatria Medica E Chirurgica: Medical and Surgical. Pediatrics. (2017) 9:155. 10.4081/pmc.2017.155 28673076

[5] Green CK Shurley T. What's in the bottle? a review of infant formulas. Nutr Clin Pract. (2016) 31:723–9. 10.1177/0884533616669362 27646861

[6] Clemens RA Hernell O Michaelsen KF. Human milk vs. cow's milk and the evolution of infant formulas. Nestle Nutrition Workshop. (2011) 61:17–28. 10.1159/000325572 21335987

[7] Pillai A Albersheim S Matheson J Lalari V Wei S Innis SM. Evaluation of a concentrated preterm formula as a liquid human milk fortifier in preterm babies at increased risk of feed intolerance. Nutrients. (2018) 10:1433. 10.3390/nu10101433 30287775 PMC6213423

[8] Wang CG. Effect of biological zinc and organc zinc on the growth and development of infants. China Tropical Medicine. (2009) 9:580–1.

[9] Hurrell R Egli I. Iron bioavailability and dietary reference values. Am J Clin Nutr. (2010) 91:1461–7. 10.3945/ajcn.2010.28674F 20200263

[10] Nair KM Augustine LF. Food synergies for improving bioavailability of micronutrients from plant foods. Food Chem. (2018) 238:180–5. 10.1016/j.foodchem.2016.09.115 28867091

[11] Sheftel J Loechl C Mokhtar N Tanumihardjo SA. Use of stable isotopes to evaluate bioefficacy of provitamin a carotenoids, vitamin a status, and bioavailability of iron and zinc. Adv Nutr. (2018) 9:625–36. 10.1093/advances/nmy036 30239582 PMC6140444

[12] Lou M B. Observation on the clinical effect of ferrous gluconate in the treatment of iron deficiency anemia in children. Straits Pharmaceutical. (2015) 2:183–4. 10.14033/j.cnki.cfmr.2016.33.013

[13] Jaroslav J Lenka KK Luboš S Vladimir B Petr S František S. Development of novel liquid chromatography method for clinical monitoring of vitamin B1 metabolites and B6 status in the whole blood. Talanta. (2020) 211:120702. 10.1016/j.talanta.2019.120702 32070596

[14] Rümelin A Humbert T Fauth U. Determination of alpha-tocopherol in plasma by high performance liquid chromatography with fluorescence detection and stability of alpha-tocopherol under different conditions. Drug Research. (2004) 54:376–81. 10.1055/s-0031-1296987 15344841

[15] Srogi K. Use of microwave-assisted digestion for determination of element contents in environmental materials by ICP-AES and FAAS. Mol Plant Breeding. (2006) 19:85–6. 10.1556/APH.19.2004.1-2.12

[16] WHO and FAO. Book review: vitamin and mineral requirements in human nutrition. Food Nutr Bull. (2006) 1:82. 10.1177/156482650602700117

[17] Bocquet A Briend A Chouraqui J P Darmaun D Feillet F Frelut M. The new European regulatory framework for infant and follow-on formulas: Comments from the Committee of Nutrition of the French Society of Pediatrics (CN-SFP). Archives de Pédiatrie. (2020) 27:351–3. 10.1016/j.arcped.2020.09.002 33023722

[18] Poitevin E. Official methods for the determination of minerals and trace elements in infant formula and milk products: a review. J AOAC Int. (2016) 99:42–52. 10.5740/jaoacint.15-0246 26821839

[19] Chinese Society of Nutrition. Dietary Reference Intakes of Chinese Residents Book. Beijing: China Light Industry Press (2013). p. 32–134.

[20] Milačič R Ajlec D Zuliani T Žigon D Ščančar J. Determination of Zn-citrate in human milk by CIM monolithic chromatography with atomic and mass spectrometry detection. Talanta. (2012) 101:203–10. 10.1016/j.talanta.2012.09.002 23158313

[21] Wegmuller R Tay F Zeder C Brnic M Hurrell RF. Zinc absorption by young adults from supplemental zinc citrate is comparable with that from zinc gluconate and higher than from zinc oxide. J Nutr. (2014) 144:132–6. 10.3945/jn.113.181487 24259556 PMC3901420

[22] Cui DY. Development of pasteurized infant formula milk. Northeast Agricultural University Dissertation for the Master Degree. (2019)

[23] Xiao M. Pharmacodyrnamics and bioavailability of Zinc citrate in humans. J North Pharm. (2008) 4:26–7.

[24] Guo CH Chen P C Yeh MS Hsiung DY Wang CL. Cu/Zn ratios are associated with nutritional status, oxidative stress, inflammation, and immune abnormalities in patients on peritoneal dialysis. Clin Bioch. (2011) 44:275–80. 10.1016/j.clinbiochem.2010.12.017 21223959

[25] Sahin H Uyanik F Inanç N Erdem O. Serum Zinc, plasma ghrelin, leptin levels, selected biochemical parameters and nutritional status in malnourished hemodialysis patients. Biol Trace Elem Res. (2009) 3:191–9. 10.1007/s12011-008-8238-0 18953507

[26] Hennet T Borsig L. Breast milk at Tiffffany's. Trends Biochem Sci. (2016) 41:508–18. 10.1016/j.tibs.2016.02.008 27093946

[27] Debier C Larondelle Y. Vitamins A and E: metabolism, roles and transfer to offspring. Brit J Nutr. (2005) 93:153–74. 10.1079/BJN20041308 15788108

[28] Oftedal OT. The evolution of milk secretion and its ancient origins. Animal. (2012) 6:355–68. 10.1017/S1751731111001935 22436214

[29] Barone GO Regan J Kelly A L O'Mahony JA. Calcium fortification of a model infant milk formula system using soluble and insoluble calcium salts. Int Dairy. (2020) 117:104951. 10.1016/j.idairyj.2020.104951

[30] Allen L De Benoist B Dary O Hurrell R. Food fortification: basic principles in guidelines on food fortification with micronutrients. Rome, Italy: FAO. Report. (2006)

[31] Donna T. Understanding infant formula. Paediatr Int Child H. (2019) 29:384–8. 10.1016/j.paed.2019.06.003

[32] Saxena J Adhikari B Brkljaca R Huppertz T Zisu B Chandrapala J. Effect of compositional variation on physico-chemical and structural changes in infant formula during storage. Int Dairy. (2020) 116:104957. 10.1016/j.idairyj.2020.104957

